# Determinants of Youth Exposure to Nicotine-Containing Aerosols: Findings from a College Survey

**DOI:** 10.3390/jox16010003

**Published:** 2025-12-19

**Authors:** Chesmi Kumbalatara, Lindsey Johnson, Matthew MacArthur, Meungguk Park, Wasantha Jayawardene

**Affiliations:** 1 School of Human Sciences, College of Health and Human Sciences, Southern Illinois University, Carbondale, IL 62901, USA; chesmi.kumbalatara@siu.edu (C.K.); parkm@siu.edu (M.P.); 2 School of Medicine, Southern Illinois University, Springfield, IL 62794, USA; lindsey.johnson@siu.edu (L.J.); matthew.macarthur@siu.edu (M.M.)

**Keywords:** youth vaping, college students, electronic nicotine delivery systems (ENDS), substance use

## Abstract

Electronic Nicotine Delivery Systems (ENDSs) expose users to nicotine, volatile organic chemicals, and ultrafine particles that pose emerging toxicological concerns for youth. The prevalence of vaping among college students quadrupled between 2017 and 2019. The Vaping Initiation, Continuation, Termination, or Resumption in Youth (VICTORY) study explored a random sample of 543 undergraduate students at a Midwestern university, using an anonymous online survey, for factors associated with initiation and regular inhalation of vape-derived aerosols. Results showed that 50% of participants had ever used a vape, and 67% had used tobacco, vape, or marijuana. The mean age of first use of tobacco was 15.16 years, significantly younger than the mean ages for vaping (16.33) and marijuana (16.60). There were no significant gender differences in ENDS use, although more males reported tobacco as their first substance (18% difference). Notably, 40% reported non-alcoholic substance or alcohol use in the past 30 days. Decision-tree analysis revealed complex relationships between vaping aerosols, tobacco, alcohol use, marijuana use, and living arrangements. Logistic regression identified key predictors of regular vaping, including higher school year, lower household income, employment status, and younger age at first use. These findings highlight the need for tailored public health interventions and continued monitoring to address the growing trend of youth vaping.

## 1. Introduction

Electronic nicotine delivery systems (ENDSs) have become the most widely used tobacco products among U.S. youth, encompassing vapes, vaporizers, vape pens, e-cigarettes, e-cigars, hookah pens, and e-pipes [1]. While ENDSs are often marketed and perceived as less harmful than combustible cigarettes, their aerosol still contains toxicants, including nicotine, carcinogenic agents, heavy metals, and volatile organic compounds [2]. These risks were highlighted by the 2019–2020 EVALI outbreak, which resulted in over 2800 hospitalizations and 68 deaths due to vitamin E acetate exposure [3]. Nicotine, in particular, poses unique risks for adolescents, as it impairs cognitive function, memory, and executive functioning during critical periods of brain development [4] and ENDS use had a positive association with combustible cigarette use for cancer survivors [5]. In the meantime, new, more attractive ENDS products are flooding the market at an alarming rate, along with marketing strategies that are advancing much faster than public health programming. New vape devices offer substantially higher, more addictive concentrations of nicotine. For example, some popular pod-based e-cigs contain 50 mg of nicotine, which is equal to the total amount of nicotine in a pack of regular cigarettes [6].

Despite these harms, youth uptake of ENDSs has increased dramatically in recent years, even as cigarette smoking rates have continued to decline. According to the 2023 National Youth Tobacco Survey (NYTS), 10.0% of U.S. high school students currently use e-cigarettes, with disposable flavored products remaining the dominant choice [7]. Similarly, the 2024 Monitoring the Future (MTF) survey found that 21.0% of 12th graders and 15.5% of 10th graders reported past-year nicotine vaping [8]. To effectively address the increase in ENDS use, it is essential to explore the factors that influence ENDS use and the sequence of their occurrence.

Studies suggest that many youth who vape report ENDS as their first tobacco product, while far fewer report cigarettes as their initiation point [9]. This pattern raises concerns that ENDS may reverse decades of progress in tobacco control by renormalizing nicotine use among younger generations. Empirical studies found that, among adolescents who vape in high school, stress relief was the most cited reason for vaping, followed by peer influence [10]. Furthermore, living conditions and family history of smoking are well recognized to be strong determinants of adolescent ENDS product uptake [11]. It is also important to recognize the psychosocial and behavioral factors that can cause an increase in ENDS products use [12].

While public health campaigns and educational programs have strongly emphasized the dangers of combustible cigarettes, many youth perceive vaping as a harm reduction strategy, because comparable messaging about the risks of ENDS use has lagged behind, leaving adolescents less informed about vaping harms [13]. Moreover, the scientific evidence on long-term ENDS health risks remains less definitive than that of smoking, further contributing to increased use of ENDS [14]. Consequently, youth may view vaping as a safer or more socially acceptable alternative, despite mounting evidence of its harms.

The present study examined factors associated with lifetime and self-reported regular ENDS use among undergraduate students, with the goal of informing prevention strategies and policy considerations that reflect these distinct dynamics.

## 2. Materials and Methods

Vaping Initiation, Continuation, Termination, or Resumption in Youth (VICTORY) was a study funded internally to promote research activities among undergraduate students in health and human sciences. Undergraduate research assistants, guided by a faculty principal investigator, contacted and surveyed fellow students, designed questions, increased response rates, collected responses, and helped report results, which received approval from the Institutional Review Board. The study employed an exploratory cross-sectional online survey to examine vaping behaviors among students at a Midwestern university.

### 2.1. Research Site Description

There were 11,107 students enrolled during the 2022–2023 academic year in the university where the research took place. Among these, there were 8000 undergraduate students, with 83.9% full-time and 80.5% residing on-campus. Almost all the students (98.3%) in the college were from the United States. Most of them (83.7%) came from the same state as the college. The university is situated in a rural county and region.

### 2.2. Study Population

The study comprised both full-time and part-time students attending educational sessions during the spring or summer of 2023, either on campus or in remote learning settings. Vaping status was not a determinative factor in this investigation. Therefore, the study encompassed individuals who were current or former users of ENDS products as well as those who had never used them. The informed consent documentation explicitly excluded individuals under the age of 18 at the time of the survey from participating in the study.

### 2.3. Sampling

Emails introducing the study were dispatched randomly to a targeted student body in both February 2023 for 4000 and June 2023 for 1000. Each email contained a detailed message and an attached information flyer elucidating the study’s objectives. Those expressing interest were encouraged to access additional information and indicate their consent by either clicking on a provided hyperlink or scanning a QR code that directed to a Qualtrics survey. Reminder emails were dispatched a week later and subsequently two weeks after the initial invitation. The study enrollment period spanned approximately four weeks. While the study did not broadly solicit participation to avoid convenience sampling, the potential for participants to disseminate information about the study to their acquaintances could not be excluded.

### 2.4. Instrument

The Qualtrics survey incorporated skip logics, enabling respondents to bypass specific questions based on their previous responses. The survey comprised 48 structured questions, necessitating respondents to select answers from a provided list, along with a few open-ended questions for voluntary written responses. The questions encompassed various formats (Likert-type, multiple-selection), including scales with slider bars for selection. The survey was tested on computer and smartphone platforms before deployment, with most respondents taking about eight minutes to complete.

If a participant reported no prior vaping experience, they were not required to respond to further inquiries related to vaping practices. Instead, they were invited to provide general personal information such as their place of residence. Additionally, the survey included queries about their tobacco, alcohol, and marijuana use, as well as inquiries about acquaintances who vape. Furthermore, participants who acknowledged a history of vaping experimentation without regular engagement were asked about their initial experience with vaping and whether they presently continue this practice.

### 2.5. Statistical Analysis

Data analysis was conducted using IBM SPSS statistical software version 29 [15]. The descriptive analysis was performed for all variables related to ever-vapers and regular vapers with mean and standard deviation calculated for continuous variables; frequencies and percentages were calculated for categorical variables. The sample was predominantly composed of White participants; therefore, the other racial groups were combined for analysis. To examine patterns of initiation, a variable representing the first use of a non-alcoholic substance was derived. Among participants who reported an age of first use for at least one non-alcoholic substance (*n* = 360), those who provided the same minimum age for two or more substances (*n* = 68) were excluded, as the sequence of initiation could not be determined. The final analytic sample consisted of 292 participants, whose first reported non-alcoholic substance was vape (*n* = 103), tobacco (*n* = 81), or marijuana (*n* = 108).

Decision-tree analysis employed decision-tree classifiers to create optimal decision-tree models that effectively represented the distinct subgroups that were identified [16]. Analysis was conducted to classify participants by vaping status (ever vs. never use) based on demographic and substance-use variables, including alcohol-related factors, in a sample of 543 individuals. For this analysis, the CHAID (Chi-squared Automatic Interaction Detection) method was used in IBM SPSS statistical software under decision tree analysis. The Chi-square test was used with 0.05 significance level in this method.

Binary logistic regression was conducted to classify participants as regular vapers or non-regular vapers. Prior to analysis, multicollinearity was assessed using the variance inflation factor (VIF) and found to be within acceptable limits [17]. The Shapiro–Wilk test was used to evaluate the normality of continuous variables. The model included several combinations of demographic and vaping-related variables, and the final model retained variables that were statistically significant at α = 0.05. Of the 270 participants who reported their vaping pattern, 92 cases contained missing data on one or more predictor variables and were therefore excluded using listwise deletion, resulting in 178 complete cases for the final analysis.

## 3. Results

An online survey was conducted on a random sample of 5000 university associates, with 605 individuals providing consent and 543 successfully completing it. Of the participants, 270 (50%) reported ever using a vape, and the ever-use was not significantly different between genders (males = 49.7%, females = 49.4%). At the same time, 363 (67%) participants had ever used tobacco, vape, or marijuana more than once.

Out of the 292 students who correctly identified their age at first non-alcoholic substance use, 37% reported marijuana as the first substance they used, and 35% reported vaping (Table 1). In contrast, only 28% reported tobacco as their first substance. The mean age of first use was significantly different across non-alcoholic substances: tobacco was first used at a mean age of 15.16 years (*SD* = 3.77), while vaping was first used at 16.33 years (*SD* = 2.26), and marijuana at 16.60 years (*SD* = 2.26).

When broken down by gender, tobacco was the most common first non-alcoholic substance for males (39%), whereas only 22% of females reported using tobacco first. In contrast, 40% of females reported marijuana as their first non-alcoholic substance, compared to just 28% of males. Vaping was the first non-alcoholic substance for 33% of males and 38% of females. Among participants identifying as another gender, marijuana was the most common first-use non-alcoholic substance, reported by 59%. (The row percentages were not depicted in Table 1).

A total of 67% of students reported ever using one or more non-alcoholic substances. However, only 40% (219 students) have used any of these substances at least once in the past 30 days. Among those who used those substances in the past 30 days, marijuana was the most used, with 71% of participants reporting use, and an average of 14.22 days (*SD* = 11.65) of use. Vaping was used for a higher average number of days, 15.47 days (*SD* = 12.23), and was reported by 40% of respondents as part of their past 30-day substance use. In contrast, tobacco was used the fewest days in the past 30 days (*M* = 7.36, *SD* = 9.48), and only 21% of participants reported using it.

Significant differences were observed based on whether students had ever vaped. Students who had reported ever-vaped, had significantly higher past 30-day use of tobacco (*M* = 7.95, *SD* = 9.73) compared to those who had never-vaped (*M* = 1.25, *SD* = 0.50; t = 4.35, *p* < 0.001). Similarly, students who had ever-vaped also reported significantly higher past 30-day use of marijuana (*M* = 15.46, *SD* = 11.59), compared to never-vapers (*M* = 8.33, *SD* = 10.22; t = 3.22, *p* = 0.001) (Table 2). Additionally, students who had ever used tobacco reported significantly higher past 30-day vaping use (*M* = 16.75, *SD* = 12.21) compared to those who had never used tobacco (*M* = 12.19, *SD* = 11.81; t = 1.92, *p* = 0.029). However, no significant difference was found in the past 30-day use of vaping or tobacco between students who had ever used marijuana and those who had never used it.

There were slight differences in past 30-day vaping based on factors such as gender, ethnicity, race, household type, household income, and what people students live with (Table 2). However, none of these differences were statistically significant. In contrast, employment status had a significant effect on past 30-day tobacco use. Students who were not employed reported a higher mean number of days using tobacco in the past 30 days (*M* = 9.96, *SD* = 11.01) compared to those who were employed (*M* = 4.64, *SD* = 6.77), with a statistically significant difference (t = 1.96, *p* = 0.001). Among past 30-day vapers, students who agreed with the statement, “someone who has health concerns that might be caused by vaping,” reported fewer days of vaping (*M* = 13.39, *SD* = 11.79) than those who disagreed with the statement (*M* = 17.08, *SD* = 12.40). This difference was statistically significant (t = −1.71, *p* = 0.045).

**Figure 1:**
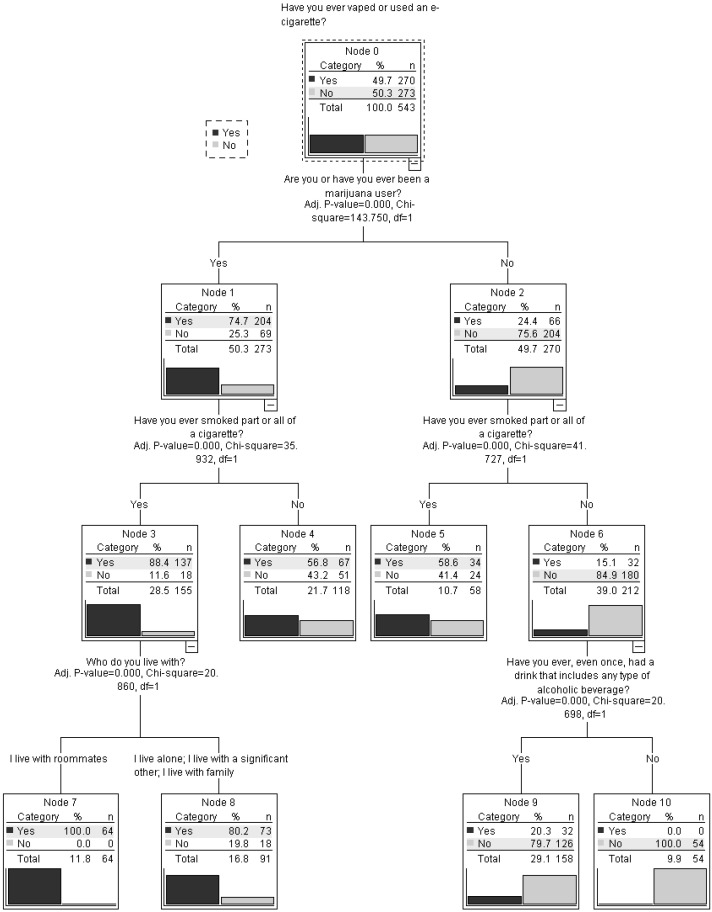
Decision Tree to Classify Ever-Vapers (*N* = 543).

The decision tree analysis (Figure 1) showed that the likelihood of students ever using a vape was influenced by their use of marijuana, tobacco, and alcohol. For students who had ever used marijuana, there was a 57% chance they had also ever used a vape, even if they had never smoked a cigarette or a part of it. The probability of ever using a vape increased to 100% if they had ever smoked a cigarette or a part of it and lived with roommates. This split reflects that, in the model, cigarette experimentation and living with roommates were the strongest distinguishing variables for this subgroup. For students who had never used marijuana, the likelihood of ever using a vape was 59% if they had ever used tobacco. However, students who had neither used marijuana nor tobacco had only a 15% chance of ever using a vape, specifically, those who had never used alcohol had a 0% chance of vaping. If these students had used alcohol, the chance of having ever vaped increased to 20%. Within the subgroup with no marijuana or tobacco use, alcohol use was the next variable selected by the model to distinguish between students who had and had not ever vaped.

The fitted binary logistic regression model (Table 3) correctly classified 73.4% of regular vapers (58 out of 79) and 78.8% of non-regular vapers (78 out of 99). The results indicated several key patterns: students in higher school years were less likely to vape regularly, with the odds of regular vaping decreasing by about 37% for each additional year of school. Students from higher-income households had lower odds of regular vaping, with the odds decreasing by approximately 56% for each one-unit increase in household income. Employed students were much more likely to vape regularly than those not working, with their odds increasing by a factor of about 35.2. Additionally, for each additional year of age when students first vaped, the odds of regular vaping increased by around 18%.

A total of 178 valid cases were included in the logistic regression model incorporating the variables presented in Table 3. The model correctly classified 73.4% of regular vapers (58 of 79) and 78.8% of non-regular vapers (78 of 99). Students in higher years of study were less likely to vape regularly, with the odds of regular vaping decreasing by approximately 37% for each one-unit increase in academic year (95% CI = [0.44, 0.90]). Participants with higher household incomes also had lower odds of vaping regularly, decreasing by about 56% with each one-unit increase in income category (% CI = [0.24, 0.82]). Employed individuals were substantially more likely to vape regularly compared to those who were not employed, with the odds increasing by a factor of approximately 35.2 (95% CI = [3.17, 390.78]). Additionally, for each additional year of age at first vaping, the odds of regular vaping increased by roughly 18% (95% CI = [1.07, 1.30]).

## 4. Discussion

This study contributes to the growing literature on ENDS use in the post-COVID-19 context by highlighting the high prevalence of vaping among college students. Half of the surveyed students reported ever using an ENDS product, exceeding rates documented in some prior studies of similar populations [18]. Importantly, our findings revealed that ENDS use was not significantly associated with gender, aligning with prior work suggesting that vaping behavior may be less gender-stratified than smoking [19]. A study performed by Dawkins and colleagues found that females preferred sweet flavors of ENDS product liquids and brands that closely resembled tobacco cigarettes in appearance and preferences that corresponded to more positive perceptions of taste and their ability to reduce nicotine cravings [20]. Instead, factors such as age, household income, and early initiation were stronger predictors of regular vaping in the current study.

The fact that ENDS use surpassed both tobacco and marijuana use in the past 30 days is particularly concerning. On average, students who vaped reported use on more days per month than those using other substances. While ENDS are commonly perceived as less harmful than combustible tobacco, this perception may contribute to more frequent use and progression from experimentation to regular use [21]. Furthermore, polysubstance use patterns identified in this study, such as the co-use of ENDSs with tobacco and marijuana, raise concerns about vaping serving as both a gateway and a facilitator of broader substance involvement [22,23].

These results underscore a critical issue in current tobacco control policy: smoking and vaping are regulated under the same framework, despite clear evidence that youth are disproportionately initiating nicotine use through ENDSs. One reason may be the success of anti-smoking education campaigns in shaping youth attitudes toward combustible tobacco, while equivalent efforts regarding vaping have been slower and less comprehensive [24]. Additionally, the uncertainty surrounding long-term ENDS harms may further enable youth to downplay potential risks [14]. As a result, current policies may be insufficiently responsive to the unique appeal and widespread adoption of vaping among youth.

A differentiated policy approach may therefore be warranted. Strategies that have successfully reduced youth smoking, such as graphic warning labels, counter-marketing campaigns, and targeted taxation, could be adapted and intensified for vaping products. At the same time, policies should account for the distinct features of ENDSs, such as flavored products, discrete device designs, and online availability, that make them especially attractive to young users [25,26]. Conversely, personalized cessation programs should be designed to assist individuals who are addicted to vaping [27]. Without such adjustments, the rapid rise in vaping threatens to undermine decades of progress in reducing youth nicotine initiation.

This study is not without limitations. The first being that the survey was conducted at a mid-size university in a rural setting. Due to the differences in beliefs, values, and resources, the generalization of our findings to urban settings, other states, or countries should be done with caution. Another important limitation is that a nonresponsive bias cannot be excluded. Furthermore, a well-documented concern in youth health surveys is sincerity bias, which occurs when respondents provide answers that do not reflect their true beliefs or behaviors, either intentionally (e.g., impression management) or unintentionally due to a subconscious desire for social approval (e.g., self-deception) [28]. Additionally, the overwhelming majority of respondents were non-Hispanic White. Further testing would be needed to apply our findings to individuals of other demographics. An additional limitation is that the survey did not assess psychiatric or mental health conditions, which are known to be prevalent among university populations and are often associated with increased substance use, including vaping. Because we did not collect data on mental health status, we were unable to examine how psychiatric disorders may have influenced vaping initiation or regular use in this sample. Future research should incorporate validated mental health measures to better understand these relationships. Nonetheless, the findings highlight the urgency of reassessing current approaches to ENDS regulation. By recognizing the unique drivers of vaping among youth and developing tailored prevention strategies, policymakers and public health practitioners may be better positioned to mitigate this growing epidemic.

## 5. Conclusions

In conclusion, the rising use of ENDSs among youth is a significant public health concern, with half of the survey respondents reporting ever using in their lifetime. Social factors, such as living with roommates, were linked to higher vaping rates. The success of anti-smoking efforts, coupled with limited regulation of ENDSs, may explain this shift in behavior. To curb this trend, targeted prevention, flavor restrictions, and stricter marketing controls are needed, along with evidence-based policies addressing vaping’s unique risks.

In addition, this study highlights that information campaigns about ENDSs should go beyond reducing vaping to prevent early nicotine dependence, discouraging shifts to more harmful products, and supporting evidence-based harm-reduction approaches rather than using a strictly prohibitionist stance. Because youth often underestimate vaping risks and frequently use multiple substances, prevention efforts should also address polysubstance use, specifically early alcohol use and its known harms to brain development. Integrating messages about nicotine, alcohol, and other substances may better reduce clustered risk behaviors among young people.

## Figures and Tables

**Table 1:** Population Characteristics by First Ever Use of Non-alcoholic Substances (*N* = 292) and Alcohol (*N* = 306).

Variable		First Used Substance			
		Vape *	Tobacco *	Marijuana *	Alcohol **
Gender	*N*	101 ***	81	108	303 ***
	Male	30.69%	45.68%	24.07%	30.36%
	Female	67.33%	48.15%	66.67%	64.03%
	Other	1.98%	6.17%	9.26%	5.61%
Are you of Spanish, Hispanic, or Latino origin?	*N*	102 ***	81	108	304 ***
	Yes	5.88%	9.88%	12.04%	7.89%
	No	94.12%	90.12%	87.96%	92.11%
Race	*N*	102 ***	80 ***	106 ***	298 ***
	Other	11.76%	12.50%	22.64%	9.73%
	White	88.24%	87.50%	77.36%	90.27%
Have you ever vaped or used an e-cigarette?	*N*	103	81	108	306
	Yes	100.00%	60.49%	49.07%	43.46%
	No	0.00%	39.51%	50.93%	56.54%
Have you ever smoked part or all of a cigarette?	*N*	103	81	108	306
	Yes	45.63%	100.00%	33.33%	34.31%
	No	54.37%	0.00%	66.67%	65.69%
Are you or have you ever been a marijuana user?	*N*	103	81	108	306
	Yes	60.19%	48.15%	100.00%	40.20%
	No	39.81%	51.85%	0.00%	59.80%
Have you ever, even once, had a drink that includes any type of alcoholic beverage?	*N*	103	81	108	306
	Yes	98.06%	95.06%	97.22%	100.00%
	No	1.94%	4.94%	2.78%	0.00%

* Where records were excluded if the responder had given the same minimum age for two or more non-alcoholic substances. ** Where records were excluded if the responder had given the same minimum age for any non-alcoholic substance and alcohol. *** Missing values were observed in these crosstabs.

**Table 2:** Population Characteristics by Number of Days of Past 30 Days Non-alcoholic Substance and Alcohol Use (*N* = 394).

Variable	Category	During the Past 30 Days Used							
		Vape		Tobacco		Marijuana		Alcohol	
Demographics									
		*N*	*M* (*SD*)	*N*	*M* (*SD*)	*N*	*M* (*SD*)	*N*	*M* (*SD*)
Gender	Male	46	14.98 (12.44)	16	5.56 (6.83)	47	16.49 (11.41)	117	7.99 (7.38)
	Female	77	15.19 (12.22)	28	7.86 (10.50)	100	13.04 (11.59)	229	6.39 (5.60)
	Other	4	24.25 (9.60)	1	22.00 (0.00)	7	16.71 (13.92)	16	5.81 (7.47)
Spanish, Hispanic, or Latino origin	Yes	11	13.36 (12.10)	4	3.25 (2.63)	13	11.62 (9.66)	33	6.85 (5.48)
	No	116	15.59 (12.30)	41	7.76 (9.82)	141	14.50 (11.85)	329	6.88 (6.43)
Race	Other	17	13.47 (11.74)	1	4.00 (0.00)	26	13.81 (10.74)	39	5.10 (5.13)
	White	109	15.71 (12.41)	44	7.43 (9.57)	125	14.37 (11.90)	318	6.99 (6.34)
What type of housing do you have?	Dorm	29	13.28 (12.13)	8	5.75 (8.36)	43	11.74 (11.35)	84	5.99 (5.78)
	House	28	15.75 (13.32)	5	16.20 (13.54)	25	13.04 (12.00)	104	5.88 (5.86)
	Apartment	71	16.25 (11.89)	32	6.38 (8.59)	87	15.78 (11.58)	176	7.84 (6.73)
Who do you live with?	Alone	32	13.84 (12.88)	13	7.77 (9.19)	47	12.28 (10.83)	89	6.21 (6.25)
	With family	19	15.84 (13.50)	5	13.40 (15.19)	15	15.73 (13.40)	54	5.30 (5.29)
	With roommates	60	15.22 (11.58)	20	4.35 (6.27)	74	13.62 (11.55)	163	7.69 (6.66)
	With a significant other	17	19.00 (12.12)	7	10.86 (11.65)	19	20.16 (11.42)	58	6.95 (6.19)
Employment status (last three months)	Not working	70	14.31 (12.30)	23	9.96 (11.01)	83	13.82 (11.74)	183	6.95 (6.53)
	Working	57	16.74 (12.16)	22	4.64 (6.77)	70	14.69 (11.74)	178	6.84 (6.17)
Household income (12 months)	≥$25,000	61	14.93 (11.90)	18	9.72 (10.97)	78	12.45 (11.29)	194	6.15 (5.58)
	<$25,000	38	17.55 (12.68)	20	4.80 (7.59)	46	15.76 (11.90)	102	8.43 (7.41)
Opinions									
How much do you agree with this statement? I know someone who has health concerns that might be caused by vaping.	Yes	56	13.39 (11.79)	23	5.22 (7.25)	83	13.87 (12.18)	188	6.72 (6.19)
	No	72	17.08 (12.40)	22	9.59 (11.08)	72	14.63 (11.09)	176	6.99 (6.51)
Roughly, what percentage of undergraduate students at your university would you say vape?	Less than 50%	63	16.54 (12.48)	21	9.52 (11.14)	82	13.98 (11.53)	202	5.41 (5.47)
	More than 50%	65	14.43 (11.98)	24	5.46 (7.47)	73	14.49 (11.87)	162	8.65 (6.88)
Substance use									
Have you ever vaped or used an e-cigarette?	Yes	128	15.47 (12.23)	41	7.95 (9.73)	128	15.46 (11.59)	228	8.00 (6.74)
	No	0	0.00 (0.00)	4	1.25 (0.50)	27	8.33 (10.22)	136	4.93 (5.08)
Have you ever smoked part or all of a cigarette?	Yes	92	16.75 (12.21)	45	7.36 (9.48)	91	14.80 (11.55)	181	8.64 (7.04)
	No	36	12.19 (11.81)	0	0.00 (0.00)	64	13.39 (11.84)	183	5.09 (4.98)
Are you or have you ever been a marijuana user?	Yes	104	15.31 (12.34)	38	7.68 (10.11)	155	14.22 (11.65)	221	7.78 (6.16)
	No	24	16.17 (11.96)	7	5.57 (4.86)	0	0.00 (0.00)	143	5.42 (6.36)
Have you ever, even once, had a drink that includes any type of alcoholic beverage?	Yes	125	15.12 (12.16)	45	7.36 (9.48)	151	14.17 (11.59)	364	6.85 (6.34)
	No	3	30.00 (0.00)	0	0.00 (0.00)	4	16.25 (15.88)	0	0.00 (0.00)

**Table 3:** Binary Logistic Regression to Predict Regular Vapers and Non-regular Vapers (Reference = Non-regular Vapers); (*N* = 178).

Variable	Exp (*B*)	Lower	Upper	*p* Value
Year in school? (1 through 6; 1 = Freshman, 2 = Sophomore, 3 = Junior, 4 = Senior, 5 = Graduate, 6 = Faculty/Staff)	0.63	0.44	0.9	0.012
What was your total household income before taxes during the past 12 months? (1 through 6; 1 = Less than $25,000, 2 = $25,000–$49,999, 3 = $50,000–$74,999, 4 = $75,000–$99,999, 5 = $100,000–$149,999, 6 = $150,000 or more)	0.44	0.24	0.82	0.009
What best describes your employment status over the last three months? (Reference:0 = not working, 1 = working)	35.2	3.17	390.78	0.004
Interaction of [How old were you the first time you vaped?—Age] and [During the past 30 days, did you use a vape?]	1.18	1.07	1.3	0.001
Interaction of [Roughly, what percentage of undergraduate students at your university would you say vape? (1 through 9; 1 = 10% or less, 9 = 90% or greater)] and [During the past 30 days, did you use a vape?]	0.92	0.69	1.21	0.542
Interaction of [Roughly, what percentage of undergraduate students at your university would you say vape?] and [What was your total household income before taxes during the past 12 months?]	1.16	1.04	1.29	0.007
Interaction of [Roughly, what percentage of undergraduate students at your university would you say vape?] and [What best describes your employment status over the last three months?]	0.57	0.38	0.86	0.007
Constant	0.78			

## Data Availability

The data presented in this study are available on request from the corresponding author due to the sensitive nature of the data, which includes substance use information disclosed by participants.
